# Regulation of *ERG3*, *ERG6*, and *ERG11* Genes in Antifungal-Resistant isolates of *Candida parapsilosis*

**DOI:** 10.18869/acadpub.ibj.21.4.275

**Published:** 2017-07

**Authors:** Ensieh Lotfali, Ali Ghajari, Parivash Kordbacheh, Farideh Zaini, Hossein Mirhendi, Rasoul Mohammadi, Fatemeh Noorbakhsh, Sassan Rezaie

**Affiliations:** 1 Department of Medical Parasitology and Mycology, School of Medicine, Shahid Beheshti University of Medical Sciences, Tehran, Iran; 2 Department of Medical Parasitology and Mycology, School of Public Health, Tehran University of Medical Sciences, Tehran, Iran; 3 Department of Medical Parasitology and Mycology, School of Medicine, Infectious Disease and Tropical Medicine Research Center, Isfahan University of Medical Sciences, Isfahan, Iran; 4 Department of Biology, Faculty of Science, Islamic Azad University, Varamin-Pishva, Iran; 5 Division of Molecular Biology, Department of Medical Mycology and Parasitology, School of Public Health, Tehran University of Medical Sciences, Tehran, Iran

**Keywords:** *Candida parapsilosis*, Gene expression, Ergosterol biosynthesis

## Abstract

**Background:**

*Candida parapsilosis* is one of the five common strains of yeasts involved in invasive candidiasis. The expression analysis of sterol biosynthesis pathway genes, which are associated with resistance, can assist the better understanding of antifungal resistance mechanisms.

**Methods:**

The antifungal susceptibility of 120 clinical *C. parapsilosis* isolates was examined. The changes in the gene expression related to resistance were analyzed.

**Results:**

Eight strains were resistant to fluconazole (FLC), itraconazole (ITC), and amphotericin B (AMB). The regulation variations included increased mRNA levels of *ERG3*, *ERG6*, and ERG11 and decreased mRNA levels of *ERG3* and *ERG6* in response to FLC. *ERG11* mRNA level increases in response to ITC and AMB.

**Conclusion:**

The mechanism of resistance to azoles in *C. parapsilosis* is very similar to *C. Albicans*. This feature may help to design new treatment strategy for candidiasis

## INTRODUCTION

The rate of candidiasis among patients has increased largely in recent years. One of the five frequent yeast strains involved in invasive candidiasis is *Candida parapsilosis* that is mainly common in neonates and in catheter-associated candidemia[1].

Lanosterol 14-demethylase is a member of cytochrome P450 enzyme family that is required for the synthesis of ergosterol. This enzyme is encoded by the *ERG11* gene, and it is a target for azoles. Azoles, especially fluconazole (FLC), are the most common drugs used for the treatment of candidiasis[2].

Long-term treatment of candidiasis and the emergence of resistance to azole and polyene drugs usually result in treatment failure[3]. However, the pathway for fungal sterol biosynthesis is still a known and confirmed target for antifungal drug development. In ergosterol biosynthesis pathway, there are other genes, i.e. *ERG3* and *ERG6*, that have not yet been characterized completely.

The 14-methyl fecosterol accumulation is caused by mutations in the *ERG3* gene. *C. albicans* *erg3* mutants are able to resist both polyene and azole treatment [4]. Sterol content analysis of *erg3* mutants shows an accumulation of sterol intermediates, i.e. 14-methyl fecosterol, which led to an impairment in the final steps of the ergosterol pathway[5].

Sequencing of *ERG6* gene has indicated a specific missense mutation in *ERG6* where cysteine is replaced with phenylalanine[5].

Resistance to polyenes is less common than azoles, but it has been recently reported in Candida species[6,7].

In the present study, eight resistant strains of *C. parapsilosis* were isolated from clinical samples. Their resistance to specific antifungal agents was validated by *in vitro* susceptibility assay. Using real-time PCR method, we made an attempt to investigate the possible alterations in expression profile of some ergosterol biosynthetic genes such as *ERG3*, *ERG6*, and *ERG11* in resistant species[8].

## MATERIALS AND METHODS

### Clinical isolates

In total, 120 clinical *C. parapsilosis* isolates, obtained from a collection of clinical isolates, were recovered during an epidemiological study in three provinces of Iran (Tehran, Mazandaran, and Isfahan), between June 2009 and June 2010[9].

### Antifungal agents

FLC (Tehran Daru, Iran), amphotericin B (AMB, Sigma-Aldrich, USA) and itraconazole (ITC, Tehran Daru, Iran) were selected as drugs to be used in susceptibility tests. The stock solutions of FLC were prepared in distilled water. However, for AMB and ITC, we used DMSO. The solutions were then kept frozen at -70°C until use. Dilution of antifungal drugs was performed with RPMI 1640 medium (Invitrogen, USA) and buffered to pH 7.0 with 0.165 M morpholine propane sulfonic acid buffer as described previously (Sigma, USA)[10].

### Antifungal susceptibility testing

Reference antifungal susceptibility testing of the isolates was performed by the broth microdilution method described in Clinical and Laboratory Standards Institute (CLSI) guidelines, document M27-S3[11]. *C. parapsilosis* ATCC 22019 type strain from the American type culture collection was used as a control for antifungal susceptibility testing[11]. According to the guidelines of CLSI, concentration ranges were 0.125-64 µg/ml for FLC and 0.03-16 µg/ml for AMB and ITC[11]. The assay was carried out in 96-well round-bottom microtiter plates. Cell suspensions were prepared in RPMI 1640 medium and were adjusted to give a final inoculum concentration of about 0.5×10^3^-2.5×10^3^ cells/ml. The plates were then incubated at 35°C and read after 48 h[12]. The minimum inhibitory concentrations (MICs) were then determined from the readings and compared with a drug-free control. All tests were performed in duplicate. The MIC results were read according to the M27-S3 supplement of the CLSI Guide.

### RNA purification

Total RNA was extracted from *C. parapsilosis* cells using a commercial kit (Fermentas, EU). Yeast cells were harvested at the exponential phase of growth. *C. parapsilosis* culture was grown in sabouraud media (without antifungal drugs) at 32°C for 48 h and grown to an optical density of approximately 0.5-1.0 at 600 nm. Total RNA was extracted according to the manufacturer’s instruction.

### RT-PCR

#### First-strand cDNA synthesis

First-strand cDNA was synthesized from 0.1 ng to 5 µg of total RNA in a 20 µl reaction volume using a commercial kit (Fermentas, EU) according to the manufacturer’s instructions. Primers were designed using the Oligo Explorer (version 15) software and were listed in Table 1. The obtained PCR fragment was estimated to be 150–200 bp.

**Table 1:** Primers used in quantitative real-time PCR analysis

Gene	Primer	Sequence
*ERG11*	Forward	5’ CAG AAA AGT GGC GTT GTT GA 3’
	Reverse	5’ GCA GCA TCA CGT TTC CAA TA 3’
*ERG3*	Forward	5’ AGT GGG TGC AGT GAT ACA GT 3’
	Reverse	5’ TGC GGG TAA GAA GGT TGG TT 3’
*ERG6*	Forward	5’ AGC TAC CGT TCA TGC TCC AG 3’
	Reverse	5’ GTT CGG CAA CTT CAC GAC TG 3’

#### Real-time PCR

Real-time PCR was performed in a Step One Plus real-time PCR system (Applied Biosystems, Foster City, CA), and SYBR Premix Ex *Taq II* was used as a reagent specifically designed for intercalator-based real-time PCR. PCR reaction mixtures contained 2 µl of first strand cDNA, 10 µl SYBR green, 0.8 µl of each primer, and 6.4 µl dH_2_O to make a final volume of 20 μL. PCR was performed on a Rotor-Gene 3000 system (Corbett Life Sciences, Sydney, Australia) with a preliminary hold at 94°C for 30 s as initial denaturation step, followed by the 45 cycles PCR step consisting of 95°C for 50 s, 58°C for 20 s and 72°C for 30 s. Final holding was performed at 72°C for 1 min, and melting step was performed at 65-99°C.

To quantify the possible changes in *ERG3*, *ERG6*, and *ERG11* genes expression levels in *C. Parapsilosis*, RT PCR was performed. *ERG3*, *ERG6*, *ERG11* genes expression were normalized to the housekeeping gene, *ACT1*, and analyzed by using REST© software (2008, v. 2.0.7). The software uses the comparative Ct method (ΔΔCt) to analyse the data. A sensitive strain (positive control) of *C. parapsilosis* was included in each run of the experiment as a positive control. Experiments under each condition were performed in duplicate, and each experiment was repeated twice on two different days to assess the reproducibility[13].

## RESULTS

### Determination of MIC

Evaluation of the antifungal susceptibility tests showed that three (2.5%) isolates of *C. Parapsilosis* strains were resistant to FLC (FLC_R1_, FLC_R2_, FLC_R3_: MIC≥8 µg/ml). In addition, three (2.5%) and two (1.66%) isolates indicated resistance to ITC (ITC_R1_, ITC_R2_, ITC_R3_: MIC≥1 µg/ml) and AMB (AMB_R1_, AMB_R2_: MIC≥1µg/ml), respectively. There was no cross-resistance to drugs between the eight strains.

### Expression analysis of ERG3, ERG6, and ERG11 genes using ΔΔCt method

**Fig. 1:**
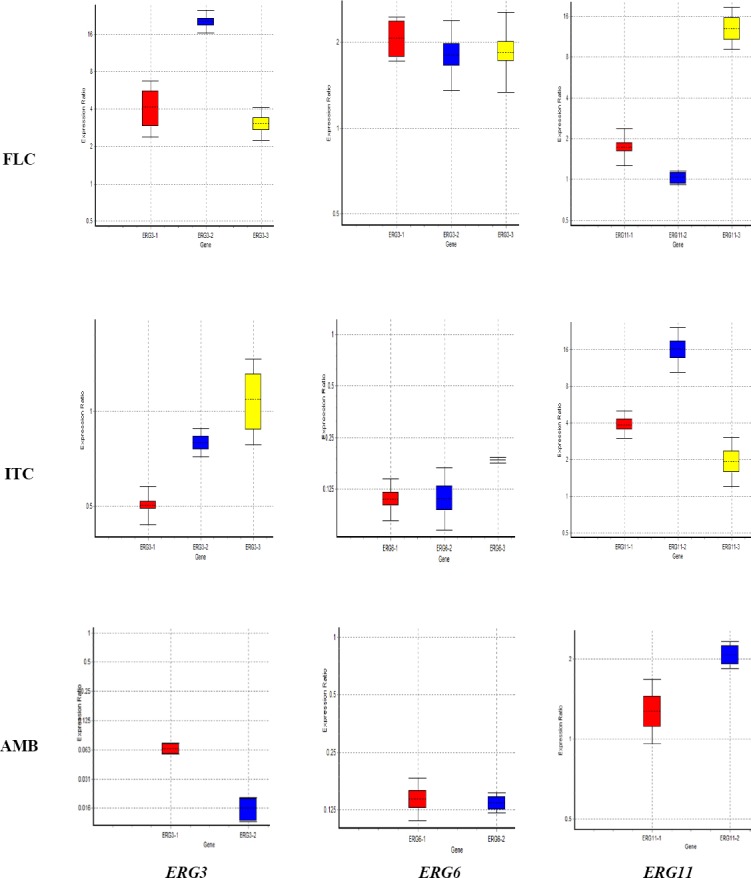
Effect of fluconazole (FLC), itraconazole (ITC), and amphotericin B (AMB) on *ERG3, ERG6*, and *ERG11* genes expression. Colors represent resistant isolates (Red, no. 1; blue, no. 2; yellow, no. 3). Results of relative expression of *ERG3, ERG6*, and *ERG11* genes was calculated using ΔΔCt method (REST©, 2008, v2.0.7. Boxes represent the interquartile range, or the middle 50% of observations. The dotted lines signify the median gene expression.

*ERG3, ERG6, ERG11*, and *ACT1* (housekeeping gene) mRNA levels were examined in all resistant strains (FLC_R1_, FLC_R2_, FLC_R3_, ITR_R1_, ITR_R2_, ITR_R3_, AMB_R1_, and AMB_R2_). The output of REST© software (2008, v. 2.0.7) was calculated for indication of *ERG3, ERG6*, and *ERG11* gene expression in the treated cells after the normalization of their expression to the housekeeping gene in all strains. Table 2 shows the results of data analysis using REST© software (2008, v. 2.0.7). Figure 1 indicates the relative gene expression level of *ERG3*, *ERG6*, and *ERG11*.

**Table 2:** Results for relative expression of *ERG3*, *ERG6*, and *ERG11* genes by use of ΔΔCt method (REST©, 2008, v. 2.0.7)

	Gene	Resistance strain	Type	Expression	P(H1)	Result
Effect of FLC	*ERG3*	FLC_R1_	TRG	3.986	0.000	Up
		FLC_R2_	TRG	20.393	0.000	Up
		FLC_R3_	TRG	3.031	0.175	-
	*ERG6*	FLC_R1_	TRG	2.056	0.000	Up
		FLC_R2_	TRG	1.803	0.000	Up
		FLC_R3_	TRG	1.847	0.166	-
	*ERG11*	FLC_R1_	TRG	1.729	0.170	-
		FLC_R2_	TRG	1.028	0.837	-
		FLC_R3_	TRG	12.951	0.000	Up
Effect of ITC	*ERG3*	ITC_R1_	TRG	0.503	0.339	-
		ITC_R2_	TRG	0.796	0.000	Down
		ITC_R3_	TRG	1.072	1.000	-
	*ERG6*	ITC_R1_	TRG	0.108	0.000	Down
		ITC_R2_	TRG	0.109	0.182	-
		ITC_R3_	TRG	0.184	0.000	Down
	*ERG11*	ITC_R1_	TRG	3.864	0.000	Up
		ITC_R2_	TRG	15.945	0.000	Up
		ITC_R3_	TRG	1.905	0.000	Up
Effect of AMB	*ERG3*	AMB_R1_	TRG	0.064	0.000	Down
		AMB_R2_	TRG	0.015	0.000	Down
	*ERG6*	AMB_R1_	TRG	0.142	0.000	Down
		AMB_R2_	TRG	0.135	0.000	Down
	*ERG11*	AMB_R1_	TRG	1.270	0.328	-
		AMB_R2_	TRG	2.071	0.000	Up
*Beta Act*		REF	REF	-	-	REF
Positive. control				-	-	1.000

Up-regulation (UP) and down-regulation (Down) for *ERG* genes. REF, reference gene; TRG, target gene; FLC, fluconazole; ITC, itraconazole; AMB, amphotericin B

## DISCUSSION

Infections due to *C. parapsilosis* have been reported in European and Asian countries and Latin America[10]. Azoles, especially FLC, are the most widely used drugs for the treatment of candidiasis. However, due to the high use of azole antifungals, the incidence of resistant strains of *Candida* has been increased[14-18]. *C. parapsilosis* is not prone to development of antifungal resistance, but recent reports indicated its increased resistance to azoles[10,19-21]. In the present study, we tried to look into the possible changes in the expression profile of *ERG3, ERG6*, and *ERG11* genes in response to FLC, ITC, and AMB within eight resistance isolates of *C. parapsilosis*. In three isolates that showed resistance to ITC, we found a significant decrease in *ERG3* mRNA level in ITC_R2_. However, mRNA levels of *ERG6* were decreased in ITC_R1_ and ITC_R3_ isolates. Surprisingly, *ERG11* mRNA levels increased in all mentioned isolates, i.e., ITC_R1_, ITC_R2_, and ITC_R3_.

Further analysis showed different expressions involved in the development of resistance to FLC among three isolates: increased mRNA levels of *ERG3* (in FLC_R1_ and FLCR_2_), of *ERG6* (in FLC_R1_ and FLC_R2_), and of *ERG11* (only in FLC_R3_). Morio[22] indicated genetic alterations in *ERG3* that may have resulted from f FLC therapy. Berkow *et al*.[23] found mutations in the sequence of the sterol biosynthesis genes (*ERG3* and *ERG11*). Based on their findings, azole resistance contributes to *MDR1* and *CDR1* (putative drug transporters). Their findings also demonstrated that among azole-resistant isolates, *Y132F* substitution in *ERG11* is the only substitution. Also, mutation in *ERG3* allows the fungal cell to produce toxic intermediate sterols and to become resistant to azoles and AMB[23]. Other investigations have shown that experimental increase in *ERG11* level can cause increased azole resistance[24,25]. In addition, drug resistance to antifungals may be regulated by transcription factors[26].

Resistance to the polyenes is rare but could be acquired by the loss-of-function mutations in *ERG3*, which can inhibit the formation of the drug-lipid complex, prevent osmotic cellular lysis and finally block the production of ergosterol. Mutations in *ERG6* led to the accumulation of last sterol intermediates and reduced susceptibility to the polyenes in *C. glabrata*[5,27].

Based on our results, AMB_R1_ and AMB_R2_ isolates showed a decrease in mRNA level of *ERG3* and *ERG6*, but AMB_R2_ isolate revealed an increase in *ERG11* mRNA level. Therefore, we can conclude that the regulation of ERG3 and ERG6 and ERG11 genes could be different in the investigated isolates.

Lees *et al*.[28] found that ERG11 (lanosterol demethylase) is essential for aerobic growth but is suppressed by mutations in the *ERG3* gene, which is in accordance with our obtained results. Silva *et al*.[14] reported that *C. parapsilosis*, like *C. albicans*, acquires resistance to azoles either through increased expression of the sterol biosynthetic pathway genes or *via* the up-regulation of the *MDR1* multidrug transporter family. In Silva’s study[14], the expression of *ERG3* and *ERG11* was reduced in FLC_R_ (-4.86 and -2.69fold), whereas in our study, the expression of *ERG3*, *ERG6*, and *ERG11* was increased or remained unchanged in FLC_RS_ (from +1.028 to + 20.39). Morio *et al*.[22] suggested more extensive investigations on other genes, such as *ERG3* and *ERG6*, which are involved in the ergosterol biosynthesis pathway, when azole resistance is suspected. Vandeputte *et al*.[7] showed that a nonsense mutation detected in the *ERG6* gene led to a decrease in ergosterol content in *C. glabrata* isolates. Expression of *ERG11* and *ERG3* genes was decreased upon exposure to AMB. Liu *et al*.[8] observed that ketoconazole increases the expression of genes involved in sterol metabolism, lipids, and fatty acid, including *ERG3* and *ERG11*. Similar to those findings, our results revealed that *ERG3* and *ERG6* genes were down-regulated due to exposure to AMB.

In summary, we can conclude that the mechanisms of resistance to azole drugs in *C. parapsilosis* and in *C. albicans* are the same. In addition, this finding may help in designing new strategies for antifungal therapy in *Candida* infections. However, further analysis is needed to determine the process by which mRNA levels for *ERG3* and *ERG6*, as well as *ERG11* are altered in these isolates.
